# Tricuspid Annular Systolic Velocity to Left Ventricular Outflow Velocity Time Integral Ratio: Proof of Concept Utility Analysis

**DOI:** 10.7759/cureus.18860

**Published:** 2021-10-18

**Authors:** Angel López-Candales, Srikanth Vallurupalli

**Affiliations:** 1 Cardiovascular Medicine, University of Missouri - Kansas City, Kansas City, USA; 2 Cardiovascular Medicine, University of Arkansas for Medical Sciences, Little Rock, USA

**Keywords:** right ventricle, pulmonary hypertension, left ventricular failure, doppler echo, echo cardiogram

## Abstract

Background

The tricuspid annular plane systolic excursion (TAPSE) / pulmonary artery systolic pressure (PASP) ratio has been a useful marker of right ventricular (RV)-pulmonary artery coupling. However, given the intricate functional and mechanical interdependence of the right and left ventricles, we believe this ratio would be less useful when assessing reduced left ventricular (LV) systolic function. Instead, we proposed using the tricuspid annular tissue Doppler imaging systolic velocity to LV outflow tract velocity time integral ratio (TA TDI s’ / LVOT VTI r) for this purpose.

Methods

For this proof-of-concept study, a retrospective analysis was conducted on 60 patients with complete echocardiographic studies while in sinus rhythm. The population was divided as follows; Group 1 included 20 individuals with normal left ventricular ejection fraction (LVEF) as well as normal RV and PASP. Group 2 was composed of 20 patients known to have been evaluated or treated for pulmonary hypertension, while group 3 was comprised of 20 patients treated for heart failure (HF).

Results

TAPSE/PASP ratios were no different from any of the studied groups. However, the proposed TA TDI s' /LVOT VTI r was statistically different among all three groups (Group 1: 0.6 ± 0.1*; Group 2: 0.5 ± 0.1°; and Group 3: 0.8 ± 0.3^#^; p < 0.001).

Conclusions

Based on these results, there is now a need for additional prospective studies to explore the overall utility of using this TA TDI s' / LVOT VTI r in day-to-day routine assessments not only for diagnostic purposes but also to determine how this ratio correlates with symptoms and changes with therapy.

## Introduction

Though invasive hemodynamic data is the gold standard to diagnose and characterize elevated pulmonary artery systolic pressures (PASP), echocardiography remains the most useful noninvasive tool for initial identification and routine follow-up [1].

Previously, PASP was mainly determined using continuous-wave Doppler determination of pressures using the maximal tricuspid regurgitation velocity [2-4]. However, significant overestimation and underestimation can occur using this approach, depending on the individual clinical situation, and consequently, levels of agreement between noninvasive and invasive assessments could be poor [5]. Therefore, it has been proposed that, instead, echocardiographic assessment of pulmonary hypertension (PH) should be limited to determining the probability of having elevated pulmonary pressures rather than estimating PASP [5]. To that end, several secondary key echocardiographic remodeling abnormalities seen as a result of elevation in PASP have been well-described in both right atria and ventricles as useful criteria suggestive of changes attributed to PH [6].

In addition to these anatomical changes, there has been interest in a recently derived noninvasive echocardiographic which measures tricuspid annular plane systolic excursion (TAPSE) / PASP rate [7]. This derived variable has been shown to be a useful marker of right ventricular (RV)-pulmonary artery (PA) coupling when compared to other right heart echocardiographic measures [7-9].

However, given the intricate functional and mechanical interdependence of the right and left ventricles [10], we believe this TAPSE/PASP rate would be less useful when assessing patients with reduced left ventricular (LV) systolic function. We based our assumption based on the fact that this TAPSE/PASP rate simply takes into account the effects of right sided-interactions, namely RV systolic function based on TAPSE and pulmonary pressures using PASP without taking into account no measure of either LV ejection fraction or overall LV ejection performance [7, 8].

From this mechanistic point of view, we propose to use the tricuspid annular tissue Doppler imaging systolic velocity to LV outflow tract velocity time integral ratio (TA TDI s’ / LVOT VTI r). This novel echocardiographic parameter not only takes into account a more useful and utilized variable of RV systolic function (tissue Doppler imaging (TDI) velocity of annular excursion) but also a more direct and efficient measure of LV performance commonly used to assess stroke volume [11- 13].

Therefore, in this proof of concept study, we tested our hypothesis that the TA TDI s’ / LVOT VTI r would be more useful in discriminating patients presenting with heart failure (HF) symptoms rather than the well-described TAPSE/PASP ratio. Therefore, to accomplish our goal, we would use the ratio of the RV outflow tract velocity time integral (RVOT VTI) to the left ventricle outflow tract velocity time integral (LVOT VTI) for our discriminatory assessment based on the intricate functional and mechanical interdependence between the right and left ventricles [6, 13-16].

## Materials and methods

Patient and data collection

Patients who were referred to our university hospital, the University of Arkansas for Medical Center (UAMS) in Little Rock, AR, for a regular transthoracic echocardiogram were considered as potential candidates for inclusion in the final analysis. All patients must have had a complete echocardiogram that included pulsed Doppler spectral signals across the RV outflow tract (RVOT), discernible tricuspid regurgitation signal, good endocardial border resolution of both the right and left ventricular chambers, as well as M-mode and TDI of the tricuspid annulus with a good TDI signal of the mitral annulus (MA). 

For this proof of concept study, we conducted a retrospective analysis of a selected group of 60 patients from our echocardiographic database. All patients were in sinus rhythm with heart rates below 100 beats per minute without any ectopy at the time of the study. Finally, studies with varying degrees of PASP and LVEF were included in the final analysis. 

Our Institutional UAMS Review Board approved this study. No consent form was required as this was a retrospective study.

Echocardiography

Two-dimensional echocardiographic studies were performed using commercially available systems (Vivid 7 and 9, GE Medical Systems, Milwaukee, Wisconsin). Images were obtained in the parasternal and apical views with the patient in the left lateral decubitus position and in the subcostal view with the patient in the supine position using a 3.5-MHz transducer. Standard two-dimensional, color, pulsed, and continuous-wave Doppler data were digitally acquired in gently held end-expiration and saved in regular cine loop format for subsequent offline analysis (EchoPAC version 111.0.00; GE-Vingmed Ultrasound AS, Milwaukee, Wisconsin). 

First, TAPSE was determined by measuring total excursion of the tricuspid annulus, from its highest position after atrial ascent to its maximal descent during ventricular systole, from the apical four-chamber view by aligning the M-mode cursor along with the movement of the lateral tricuspid annulus [6-11]. Measurements of tricuspid annular (TA) systolic velocity were then obtained using tissue Doppler imaging, as previously described, using the same anatomical orientation as for M-mode [6, 11]. 

Second, continuous-wave Doppler was utilized to record the tricuspid regurgitation jet from multiple windows, and the highest velocity was then used to estimate PASP using the modified Bernoulli equation and an estimate of mean right atrial pressure using the diameter and collapse index of the inferior vena cava and the hepatic venous flow pattern [6-11].

Calculation of the echocardiography-derived TAPSE / PASP ratio was then performed as previously described [7, 8].

Third, interrogation of the LV outflow tract (LVOT) was performed by placing a 1 to 2 mm pulsed-wave Doppler sample volume just within the LVOT from the five-chamber apical view so that the closing but not opening click of the aortic valve was visualized [14-18]. The resultant spectral Doppler signal is then traced, and the internal software package of the echocardiographic system then calculates the velocity time integral (VTI) [12, 13-16].

Fourth, measurements of MA systolic (s') and early diastolic (e') velocities were obtained using tissue Doppler imaging using the four-chamber apical view using only the lateral portion of the MA for assessment of LV systolic as well as diastolic function as previously described following guidelines published by the American Society of Echocardiography [13, 17].

Finally, interrogation of the RV outflow tract (RVOT) was performed by placing a 1 to 2 mm pulsed-wave Doppler sample volume just within the pulmonary valve from the parasternal short-axis view. The sample volume was placed so that the closing but not opening click of the pulmonary valve was visualized [6, 14-16]. RVOT VTI values were obtained by tracing the RV outflow tract spectral pulsed Doppler signals, and the echocardiographic pulmonary vascular resistance (PVR) was then calculated using a well-validated formula [6, 19].

Statistical analysis

In terms of the statistical analysis used for this study, the following steps were taken. A total of three measurements were obtained of each echocardiographic variable, and data is reported as individual mean ±standard deviation values. Baseline characteristics were compared between groups using analysis of variance (ANOVA) for continuous variables. Levene's test was first used to test to confirm the homogeneity of variances across groups, and this was followed by the Student-Newman-Keuls test that was then performed following the one-way ANOVA to compare all pairwise comparisons of the means. A stepwise multiple regression analysis was then used to identify the independent relationship between the ratio RVOT VTI / LVOT VTI and the most significant echocardiographic and Doppler variables noted in our analysis that are commonly used in the assessment of both left and right ventricular performance to determine which of these studied variables was the best discriminating predictors. Receiver operating characteristic (ROC) curves were then produced to test the ability of TA TDI s’/LVOT VTI r to identify patients with an RVOT VTI / LVOT VTI r of 0.8. A similar ROC analysis was also performed to assess the ability of the TAPSE / PASP r to identify patients using the same RVOT VTI / LVOT VTI r of 0.8. A p-value of <0.05 was considered significant. The data analysis was generated using SPSS version 17.0 (IBM Inc., Armonk, New York).

## Results

The total population of 60 patients included for analysis was obtained from a selection of consecutive studies from patients from our echocardiographic database according to their clinical profile as follows: group 1 included 20 individuals with normal LVEF as well as normal RV and PASP. Group 2 was composed of 20 patients known to have been evaluated or treated for pulmonary hypertension, while group 3 was comprised of 20 patients treated for HF. 

There was no difference in terms of either mean age (53 ± 23; 55 ± 16; 59 ± 12 years, respectively) or body surface (1.9 ± 0.2; 1.9 ± 0.3; 1.9 ± 0.2 m2, respectively) between the three groups. 

Table 1 depicts values for all collected echo-Doppler variables. A higher tricuspid regurgitation velocity with higher PAP values was seen in group 2 patients. However, PVR and TAPSE values were no different between group 2 and 3 patients. Furthermore, group 3 patients had significantly lower systolic and early diastolic tricuspid annular tissue Doppler imaging (TDI) velocity values than group 2 patients. In addition, group 3 patients also had significantly lower mitral valve E velocities, mitral annular TDI systolic and early diastolic velocities, LVOT and RVOT velocity time integral (VTI) values than group 2 patients while MV E / TDI e' ratios were significantly higher. 

**Table 1:** Echocardiographic variables for the studied population All echocardiographic variables measured in this study are shown in this table. All 60 patients had all variables measured and none were missing. PH - pulmonary hypertension, HF - heart failure, TR - tricuspid regurgitation, PASP - pulmonary artery systolic pressure, PVR - pulmonary vascular resistance, RVOT VTI - right ventricular outflow tract velocity time integral, TAPSE - tricuspid annular plane systolic excursion, TA TDI s' - tricuspid annular tissue Doppler imaging systolic velocity, MV E - mitral valve E-wave, MA TDI e' - mitral annulus tissue Doppler imaging early diastolic velocity, LVOT VTI - left ventricular outflow tract velocity time integral ratio, MA TDI s' - mitral annulus tissue Doppler imaging systolic velocity, ANOVA - analysis of variance

Variables	Control (n = 20 patients)	PH (n = 20 patients)	HF (n = 20 patients)	ANOVA
TR velocity	1.8 ± 0.5	3.4 ± 0.7	2.4 ± 0.8	P < 0.001
PASP	19 ± 8	57 ± 20	35 ± 15	P < 0.001
PVR	1.4 ± 0.7	2.8 ± 0.7	3.1 ± 1.6	P < 0.001
RVOT VTI	16 ± 3	13 ± 3	9 ± 3	P < 0.001
TAPSE	2.2 ± 0.3	1.6 ± 0.3	1.3 ± 0.4	P < 0.001
TA TDI s’	12 ± 2	10 ± 3	8 ± 3	P < 0.001
MV E	78 ± 19	102 ± 24	88 ± 25	P = 0.006
MA TDI e’	11 ± 4	9 ± 3	6 ± 2	P < 0.001
E/e’ ratio	8 ± 3	12 ± 5	18 ± 7	P < 0.001
LVOT VTI	20 ± 4	22 ± 6	11 ± 5	P < 0.001
RVOT / LVOT ratio	0.8 ± 0.2	0.6 ± 0.2	0.9 ± 0.3	P = 0.001
MA TDI s’	9 ± 2	7 ± 2	5 ± 2	P < 0.001
TAPSE / PASP ratio	1.4 ± 0.5	0.3 ± 0.1	0.5 ± 0.3	P < 0.001
TA TDI s’ / LVOT VTI ratio	0.6 ± 0.1	0.5 ± 0.1	0.8 ± 0.3	P < 0.001

**Figure 1:**
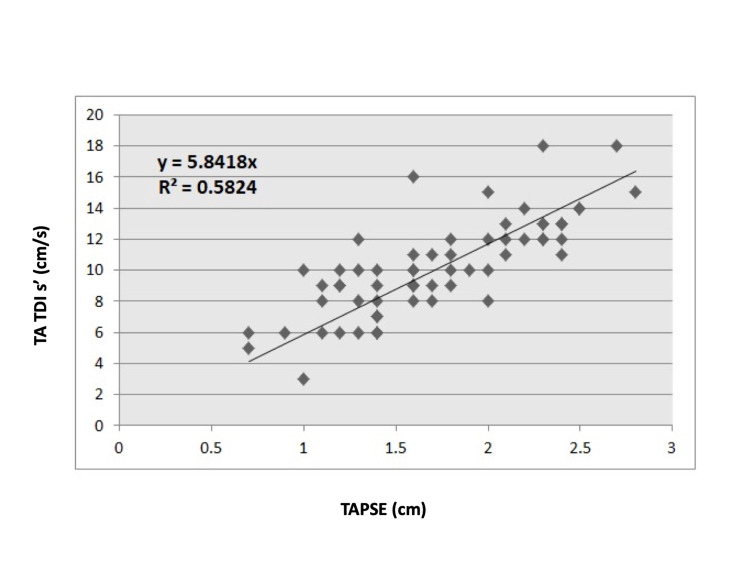
Linear regression analysis between TA TDI s' and TAPSE. A correlation analysis between the two echocardiographic variables TA TDI s' and TAPSE for our study population was performed. As shown in this figure, showed a strong correlation consistent with previously published data. This data showed that a normal expected correlation between TAPSE and TA TDI values were present to avoid cases of decoupling. TA TDI s' - tricuspid annular tissue Doppler imaging systolic velocity, TAPSE - tricuspid annular plane systolic excursion

Before analyzing the potential discriminatory ability of the TAPSE/PASP ratio, we verified that our patient population had a normally expected correlation between TAPSE and TA TDI values to avoid cases of decoupling. We accomplished this by performing a correlation analysis between the two echocardiographic variables. As shown in Figure 1, a strong correlation was seen in the study population, consistent with previously published data [6, 18, 19].

Even when the TAPSE/PASP ratio values were significantly different from group 1 when compared to both group 2 and 3; TAPSE/PASP ratios were no different from each other. Furthermore, group 3 TAPSE/PASP ratios were not even considered abnormal, based on published data [8].

**Figure 2:**
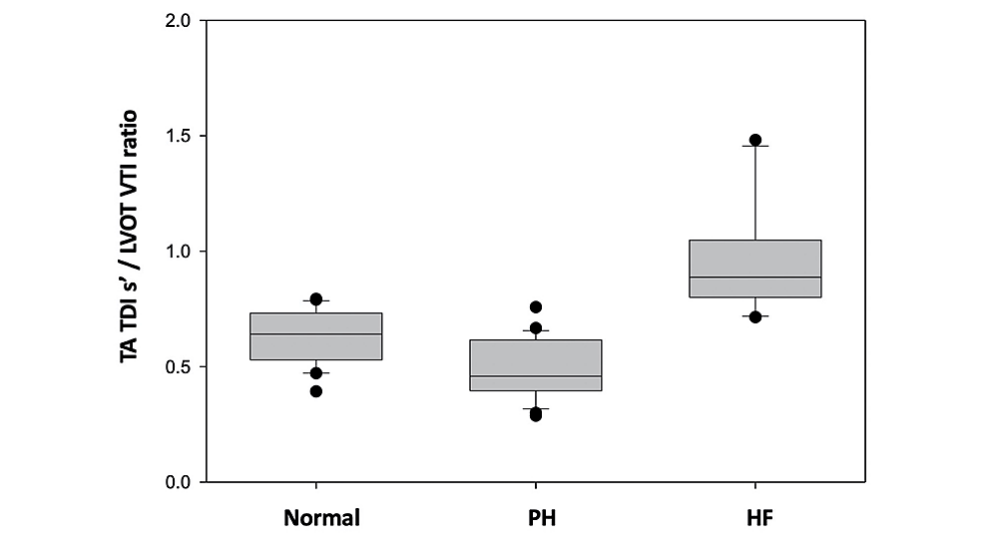
Box plot analysis Using this analysis we showed TA TDI s’ / LVOT VTI ratio for all three study groups included in this study. This calculated variable was found to be statistically different among the studied groups (group Normal: 0.6 ± 0.1; group PH: 0.5 ± 0.1; and group HF: 0.8 ± 0.3; p < 0.001). TA TDI s' - tricuspid annular tissue Doppler imaging systolic velocity, TAPSE - tricuspid annular plane systolic excursion, PH - pulmonary hypertension, HF - heart failure

In contrast, when we examined our proposed TA TDI s’ / LVOT VTI ratio it was the only calculated variable found to be statistically different among all three studied groups as shown in Figure 2. 

Based on the intricate functional and mechanical interdependence between the right and left ventricles, the ratio of the RV outflow tract velocity time integral (RVOT VTI) to the left ventricle outflow tract velocity time integral (LVOT VTI) was used as the basis for our discriminatory assessment as (RVOT VTI / LVOT VTI r). On stepwise multiple regression analysis, as shown in Table 2, the most important predictors we found useful to significantly discriminate based on RVOT VTI / LVOT VTI r were PVR, TA TDI s’, and the TA TDI s' / LVOT VTI r.

**Table 2:** Multiple regression equation Based on the intricate functional and mechanical interdependence between the right and left ventricles, we used the RVOT VTI / LVOT VTI ratio to base our discriminatory assessment. This stepwise multiple regression analysis showed that the most important predictors we found useful to significantly discriminate were PVR, TA TDI s’, and the TA TDI s' / LVOT VTI r. RVSP - right ventricular systolic pressure, PVR - pulmonary vascular resistance, TAPSE - tricuspid annular plane systolic excursion, TA TDI s' - tricuspid annular tissue Doppler imaging systolic velocity, MA TDI s' - mitral annulus tissue Doppler imaging systolic velocity, MV E - mitral valve E-wave, PASP - pulmonary artery systolic pressure, LVOT VTI - left ventricular outflow tract velocity time integral, RVOT VTI - right ventricular outflow tract velocity time integral

Independent variables	Coefficient	Std. error	p-value
RVSP	0.005662	0.001452	0.0003
PVR	-0.2085	0.02283	<0.0001
TAPSE	0.1867	0.06543	0.0062
TA TDI s’	-0.06785	0.009224	<0.0001
MA TDI s’	-0.01983	0.009669	0.0452
MV E / MA TDI e’ ratio	-0.009164	0.003048	0.0040
TAPSE / PASP ratio	-0.1120	0.05085	0.0320
TA TDI s' / LVOT VTI ratio	1.0134	0.09549	<0.0001

**Figure 3:**
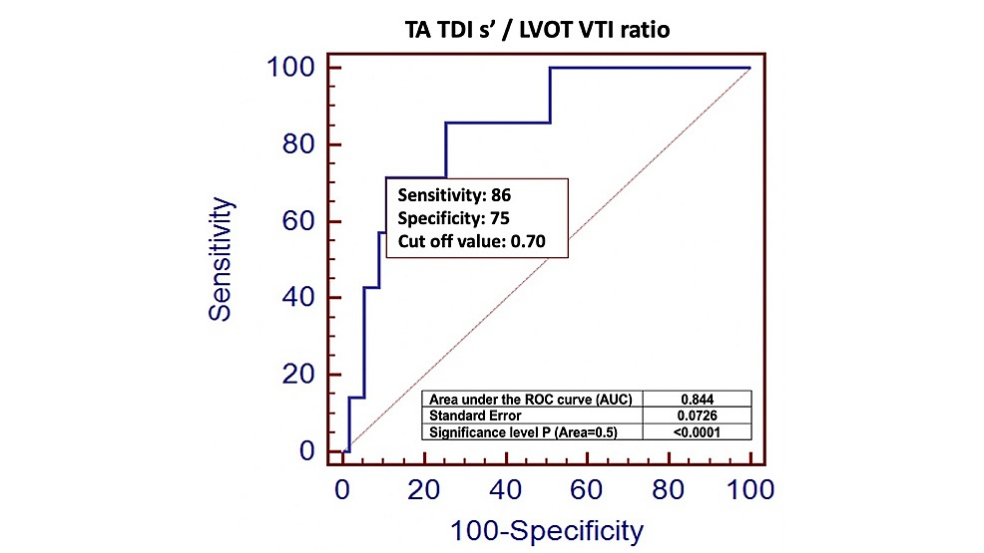
Receiver operating characteristic (ROC) curves were then produced to test the ability of TA TDI s’/LVOT VTI r to identify patients with an RVOT VTI / LVOT VTI r of 0.8. ROC curve analysis we then used to demonstrate that a TDI s' / LVOT VTI r cutoff value of 0.70 had an 86% sensitivity (95% CI: 42.1 - 99.6) and a 75% specificity (95% CI: 61.0 - 85.3) to discriminate among the study groups (area under the curve: 0.844, P <0.0001). TA TDI s' - tricuspid annular tissue Doppler imaging systolic velocity,  LVOT VTI - left ventricular outflow tract velocity time integral ratio, RVOT VTI - right ventricular outflow tract velocity time integral

Finally, as seen in Figure 3, ROC curve analysis we then used to demonstrate that a TDI s' / LVOT VTI r cutoff value of 0.70 had an 86% sensitivity (95% CI: 42.1 - 99.6) and a 75% specificity (95% CI: 61.0 - 85.3) to discriminate among the study groups (area under the curve: 0.844, P <0.0001). In contrast, the TAPSE / PASP r did poorly with a cutoff value of 0.34 had a sensitivity of only 43% (95% CI: 9.9 - 81.6), and a specificity of 72% (95% CI: 59.0 - 83.9) and was not useful to discriminate among the study groups (area under the curve: 0.504, P = 0.9).

## Discussion

The TAPSE/PASP echocardiographic ratio not only has become a meaningful prognostic parameter in patients with pulmonary artery hypertension [7, 8] but also has been advocated as a useful noninvasive echocardiographic measure of RV-PA coupling based on its ability to provide valuable information regarding RV diastolic stiffness despite not being useful as a measure of RV contractility [7-10].

However, the utility of this ratio in trying to discriminate between patients treated for PH from those affected by HF with concomitant elevation in PASP has not been previously assessed. Furthermore, the presence of elevated PASP in HF is not only clinically relevant and common but more importantly, associated with worse clinical outcomes [20, 21]. Consequently, we thought it necessary to perform this analysis using our proposed variable to test our hypothesis using complete echocardiographic data from patients treated for PH and those with HF and compared to a group of individuals with both normal LVEF and PASP. 

In our patient population, we found that this TAPSE/PASP r was not useful in distinguishing patients with PH from those patients with HF and elevated PASP. However, we found that of the studied variables, the TA TDI s’ / LVOT VTI r was statistically different among all three studied patient groups. 

Surely, it can be argued that although this ratio differed in all three groups, the absolute difference in the mean value was not much, and the standard deviation was large-the latter suggesting the possibility that a significant overlap might exist among all three groups. Therefore, we conducted a ROC curve analysis to comment on the overall diagnostic accuracy of this ratio. Based on this ROC data, the TAPSE/PASP r can as previously published[7, 8], differentiate group 1 versus groups 2 and 3 but not between the PH and HF patients. In contrast, our proposed TA TDI s’ / LVOT VTI r is able to differentiate between groups 2 and 3 patients. 

Mechanistically speaking, the intricate functional and mechanical interdependence of the right and left ventricles, the RVOT VTI / LVOT VTI r was used as the basis for our discriminatory assessment. We have already described the utility of this ratio to explain mechanical differences in terms of both interventricular dyssynchronies and in cases of PH [14-16]. In addition, the LVOT VTI is a well-known Doppler-derived measure of stroke volume obtained by measuring flow across LVOT utilizing the velocity-time integral of the Doppler signal. In fact, LVOT VTI not only is reproducible in HF but also, is superior due to its almost parallel insonation angle and relatively flat profile of velocity distribution [22, 23]. Furthermore, LVOT VTI has been shown to outperform LVEF and Doppler-derived cardiac output methods for predicting outcomes in select advanced heart failure cohorts [24]. Finally, the overall utility of TA TDI s’ for assessing RV systolic function has been extensively studied and a robust direct correlation has been seen between this longitudinal measure of RV excursion and PASP [6, 11, 18, 19].

The inability of the TAPSE/PASP r to discriminate patients as intended by our analysis might be due to a combination of factors not entirely attributed to the degree and acuity in PASP elevation of PASP but rather to the extent of RV stiffness. The latter could be in part mediated by underlying cardiomyopathic process affecting both ventricles, independent of pulmonary pressures, thus reducing its utility. Conversely, the TA TDI s’ / LVOT VTI r could be intuitively a much better echocardiographic parameter as this ratio takes into consideration LV ejection that the TAPSE/PASP r does not. Consequently, our proposed TA TDI s’ / LVOT VTI r not only utilizes a more robust variable of RV systolic function (TDI velocity of annular excursion) but also, a more direct and efficient measure of LV performance commonly used to assess stroke volume. 

Aside from the utility of these novel findings found from this pilot analysis, we recognize the following study limitations. First, the retrospective nature of the analysis, small sample size and lack of concomitant invasive hemodynamic; however, this was a proof of concept study to determine the overall utility of the easily obtained echo-Doppler variable. In addition, our results may not be generalizable to patients with arrhythmias and/or stenotic valvular disease, as they were not included in the analysis. Finally, we had no speckle tracking imaging information, which could further improve patient discrimination. However, these preliminary results using this proposed ratio in terms of patient discrimination might set a benchmark to further speckle tracking studies. 

## Conclusions

Based on these results, there is now a need for additional prospective studies to explore the overall utility of using this TA TDI s’ / LVOT VTI r in day-to-day routine assessments not only for diagnostic purposes but also to determine how this ration changes with therapy. As elegantly stated by many researchers, there is a need for additional hemodynamic variables to improve our characterization of pre-capillary PH with LV dysfunction. Our results might just be one of those variables. The opportunity for a validation study would certainly follow this proof of concept study. We foresee using this newly proposed variable not only when diagnosing or following HF patients with either reduced or preserved LVEF but also patients with either idiopathic or secondary pulmonary hypertension. We believe that the utility of this proposed echo variable would certainly be of great clinical utility on the routine, day-to-day evaluation of patients as this variable is easily obtainable and it does not require either complex measurements that would add time to the imaging study that could disturb the clinical flow of any echo lab as well as not requiring any additional off-line measurement or complex calculations.
